# Patient-reported outcomes in adequately treated hypothyroidism – insights from the German versions of ThyDQoL, ThySRQ and ThyTSQ

**DOI:** 10.1186/1477-7525-11-68

**Published:** 2013-04-23

**Authors:** Eva M Quinque, Arno Villringer, Juergen Kratzsch, Stefan Karger

**Affiliations:** 1 Max Planck Institute for Human Cognitive and Brain Sciences, Leipzig, Germany; 2 Clinic for Cognitive Neurology, University Hospital Leipzig, Leipzig, Germany; 3 Institute for Laboratory Medicine, Clinical Chemistry and Molecular Diagnostics, University Hospital Leipzig, Leipzig, Germany; 4 Clinic for Endocrinology and Nephrology, University Hospital Leipzig, Leipzig, Germany

**Keywords:** Hypothyroidism, Hashimoto’s thyroiditis, Patient-reported outcome measures (PROMs), Quality of life, Linguistic validation, Patient satisfaction

## Abstract

**Background:**

Disease-specific patient-reported outcome measures (PROMs) have been developed as important research tools in the study of various diseases. For hypothyroidism there exist three validated disease-specific questionnaires in English: the Thyroid-Dependent Quality of Life Questionnaire (ThyDQoL), the Underactive Thyroid Symptom Rating Questionnaire (ThySRQ) and the Thyroid Treatment Satisfaction Questionnaire (ThyTSQ). We report psychometric properties of new German versions of the questionnaires including construct validity from two independent samples.

**Methods:**

230 envelopes with ThyDQoL, ThySRQ and ThyTSQ were given out to patients receiving levothyroxine for diagnosed hypothyroidism. Reliability and factor analyses were performed, correlations and hypothesised subgroup differences calculated to assess psychometric properties. Independently, 18 patients with treated hypothyroidism for autoimmune thyroiditis (Hashimoto’s disease) and 18 healthy control subjects were enrolled in a clinical study. Participants filled in the above questionnaires alongside well-known generic PROMs, e.g. the Beck Depression Inventory, the 12-item Well-Being Questionnaire and the Short-Form-36. Two blood samples were taken. Groups were compared and correlations between disease-specific and generic instruments analysed. Relationships between PROMs and biochemically determined thyroid hormone status were investigated.

**Results:**

102 patients returned completed questionnaires (response rate 44%). The newly translated questionnaires had satisfactory psychometric properties. Cronbach’s alpha was 0.92 for ThyDQoL, 0.81 for ThySRQ and 0.86 for ThyTSQ. For each of the questionnaires, a single factor structure explained the data best. Adequately treated patients with thyroid stimulating hormone levels in the upper normal range reported more symptoms in the ThySRQ. Those with autoimmune hypothyroidism reported being more bothered by depressive symptoms. Within the clinical sample, correlation with well-known generic instruments revealed good construct validity. In the clinical sample patients reported more symptoms in the ThySRQ, being more bothered by tiredness, higher depression and reduced well-being despite biochemically adequate treatment. Correlations between PROMs and biochemical thyroid hormone status revealed moderate though consistent associations.

**Conclusions:**

Psychometric properties including construct validity of German versions of the ThyDQoL, ThySRQ and ThyTSQ are satisfactory. Feasibility and sensitivity in a clinical sample could be shown. We encourage the use of disease-specific PROMs in future studies as important additions to generic instruments in clinical research on hypothyroidism.

## Abstract (de)

**Zusammenfassung:**

**Hintergrund**

Krankheitsspezifische Selbsteinschätzungsfragebögen, so genannte „patient-reported outcome measures“(PROMs), wurden als wichtige Forschungsinstrumente für verschiedene Erkrankungen entwickelt. Für die Hypothyreose wurden drei englischsprachige Fragebögen entwickelt: der Lebensqualitätfragebogen zur Schilddrüsenunterfunktion (ThyDQoL), der Symptomfragebogen zur Schilddrüsenunterfunktion (ThySRQ) sowie ein Fragebogen zur Behandlungszufriedenheit bei Schilddrüsenunterfunktion (ThyTSQ). Wir stellen psychometrische Eigenschaften der neuen deutschen Versionen dieser Fragebögen vor, inklusive Daten zur Konstruktvalidität aus zwei unabhängigen Stichproben.

**Methoden**

230 Umschläge mit ThyDQoL, ThySRQ und ThyTSQ wurden an Patienten ausgegeben, die wegen einer gesicherten Hypothyreose mit Levothyroxin substituiert wurden. Mit Hilfe von Reliabilitäts- und Faktoranalysen, Korrelationsanalysen und hypothesengeleiteten Subgruppenvergleichen wurden die psychometrischen Eigenschaften der Fragebögen analysiert. 18 Patienten mit behandelter Hypothyreose autoimmuner Genese (Hashimoto Thyreoiditis) sowie 18 gesunde Kontrollprobanden nahmen an einer unabhängigen klinischen Studie teil. Die Teilnehmer füllten zu den oben genannten auch bekannte generische PROMs aus, darunter das Beck Depressions Inventar, den 12-Item Well-Being Questionnaire und den Short Form-36. Zwei Blutproben wurden entnommen. Die Gruppen wurden verglichen und Korrelationen zwischen krankheitsspezifischen und generischen Instrumenten sowie Zusammenhänge zwischen Fragebogendaten und Laborwerten berechnet.

**Ergebnisse**

102 Patienten schickten ausgefüllte Fragebögen an die Autoren zurück (Rücklaufquote 44%). Die neu übersetzten Fragebögen zeigten gute psychometrische Eigenschaften. Cronbach’s Alpha betrug 0.92 für den ThyDQoL, 0.81 für den ThySRQ und 0.86 für den ThyTSQ. Alle Fragebögen wurden am besten durch eine Einfaktorenlösung beschrieben. Patienten mit Thyroidea-stimulierendem Hormon (TSH) im oberen Normbereich berichteten mehr Symptome im ThySRQ und diejenigen mit autoimmuner Genese stärkere Beeinträchtigung durch depressive Symptome. Die Konstruktvalidität der Fragebögen konnte durch erwartungskonforme Korrelationen zu generischen Fragebögen in der klinischen Studie nachgewiesen werden. Die Patientengruppe berichtete trotz normwertigem TSH mehr Symptome im ThySRQ, mehr Beeinträchtigung durch Müdigkeit, sowie höhere Depressionswerte und geringeres Wohlbefinden als die gesunde Kontrollgruppe. Wir konnten moderate aber konsistente Zusammenhänge zwischen den Fragebogendaten und den Laborwerten finden.

**Schlussfolgerung**

Die psychometrischen Eigenschaften der Fragebögen ThyDQol, ThySRQ und ThyTSQ sowie ihre Konstruktvalidität sind zufriedenstellend. Machbarkeit und Sensitivität der Fragebögen in einer klinischen Studie konnten gezeigt werden. Die krankheitsspezifischen PROMs stellen eine wichtige Ergänzung zu den generischen Instrumenten dar, mit potentiellem Nutzen für die zukünftige klinische Forschung auf dem Gebiet der Hypothyreose.

## Background

Hypothyroidism or subclinical hypothyroidism affects 4 to 21% of the female population and 3 to 16% of the male population [1]. Standard treatment for the highly prevalent condition is replacement of thyroid hormone by levothyroxine, artificial free thyroxine (fT4) [2]. The definition of the targeted normal range of thyroid hormone level is, however, still under debate [3-5]. Moreover, it has been reported that among patients receiving this treatment, well-being is reduced even if euthyroidism is reestablished [6,7].

It is still an open issue where patients’ reports of unwanted symptoms result from [8]. Explanations discussed are independent effects of thyroid autoimmunity, the most common cause of hypothyroidism [9-11], insufficient normalisation of thyroid hormone levels at target tissues such as the brain despite normal serum hormone levels [12], selection bias in seeking health care [13] or reactive processes to the awareness of having a chronic disease [14]. Disentangling these possible causes has important implications for treatment targets in this large patient group. Crucial for successfully addressing the above issue is the use of appropriate instruments to measure patient-reported outcomes. It is important to differentiate between perceived health status, psychological well-being and quality of life as well as between generic and disease-specific instruments [15,16]. All are valid and important constructs to address patient-reported outcomes but should be carefully distinguished to avoid misleading interpretation of results. Symptom load has for example often been interpreted as quality of life although perceived symptoms may or may not influence quality of life in an individual [15,16]. Health status is often confusingly referred to as health-related quality of life [17]. It has been shown for several clinical conditions such as peripheral arterial disease or heart failure that disease-specific questionnaires are more sensitive to change [18,19]. However, most studies still use exclusively generic questionnaires and often self-constructed symptom lists to assess patient-reported outcomes in hypothyroidism, so reducing sensitivity to subtle effects and comparability across studies [20-23].

The first hypothyroidism-specific instruments have been developed and validated in recent years including the Thyroid-Dependent Quality of Life Questionnaire (ThyDQoL) [24,25]. The ThyDQoL measures the impact of hypothyroidism on quality of life in general and in selected domains tailored to the disease and to individual realities by including importance ratings for each domain. The Underactive Thyroid Symptom Rating Questionnaire (ThySRQ) is in contrast a measure of hypothyroidism-related symptoms and symptom bother [25]. Finally, the Thyroid Treatment Satisfaction Questionnaire (ThyTSQ) measures disease-specific treatment satisfaction [24,26]. It is designed to cover hypothyroidism-specific aspects such as satisfaction with current medication and dose.

All three hypothyroidism-specific questionnaires have been developed and validated in English and use of the questionnaires in any other language needs validation in an independent sample to examine psychometric validity. Although necessary, this is a demanding and time consuming procedure, possibly contributing to the paucity of validated translations. However, despite known advantages over the use of exclusively generic or non-validated instruments, the original questionnaires are also relatively new, which may account for the fact that they are not yet in widespread use. None of the validated hypothyroidism-specific questionnaires available has been evaluated for German so far, although interest in the field is high in German speaking countries [10,21-23,27].

We are thus introducing the first three hypothyroidism-specific PROMs in German to improve the array of tools available for future research. We provide detailed psychometric data including internal consistency and factor structure of the questionnaires, as well as hypothesised subgroup analyses. According to the literature we expect more negative reports in patients with thyroid stimulating hormone (TSH) in the upper normal range [28] and more negative reports in patients with hypothyroidism of autoimmune origin [10,27].

In addition, we have used the questionnaires in a clinical study including 18 adequately treated patients with hypothyroidism due to autoimmune thyroiditis (Hashimoto’s disease) and 18 healthy control subjects. The study also included a number of well-known generic PROMs. Thereby, we were able to investigate construct validity of the new questionnaires and feasibility in a clinical context. We expect disease-specific and generic instruments to be moderately correlated because similar, though distinct, constructs are targeted. The clinical study included assessment of TSH, fT4, free triiodothyronine (fT3) as well as thyroid peroxidase antibodies (TPOAb) and thyroglobulin antibodies (TgAb). This design allowed investigation of the relationship between PROMs and biochemical thyroid hormone status.

## Methods

### Linguistic validation

**Figure 1:**
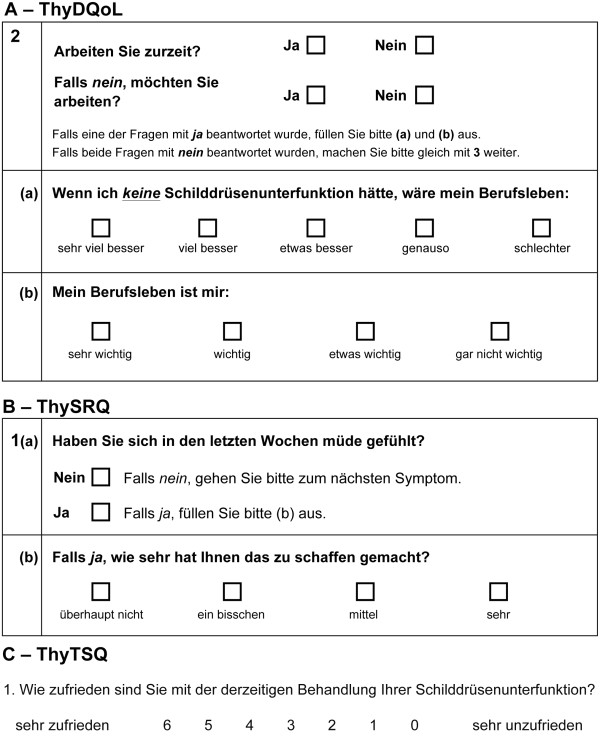
Sample items from the newly translated German versions of the (A) Thyroid-Dependent Quality of Life Questionnaire (ThyDQoL), (B) the Underactive Thyroid Symptom Rating Questionnaire (ThySRQ), and (C) the Thyroid Treatment Satisfaction Questionnaire (ThyTSQ).

Linguistic validation was performed for ThyDQoL, ThySRQ and ThyTSQ as previously described for similar instruments [29]. English originals were translated into German by two native German speakers, including a clinical endocrinologist (SK) and reconciled into a preliminary forward translation by a psychologist (EQ). The resulting forward translation was subsequently translated back into English by each of two native English speakers. Any discrepancies between original and back-translation were discussed with the developer’s team and improvements made where necessary. The resulting draft translation was then used for cognitive debriefing interviews with five patients with hypothyroidism of different origins, recruited from the volunteer database of the Max Planck Institute for Human Cognitive and Brain Sciences (MPI) and reimbursed for their time. Example items of the final German versions can be found in Figure 1.

### Scoring of questionnaires

The scoring for the newly translated questionnaires ThyDQoL, ThySRQ and ThyTSQ will be briefly summarised as explained in detail elsewhere [25,26]. The ThyDQoL starts with two overview items on present quality of life (present QoL) and impact of hypothyroidism on quality of life in general (impact on QoL). The first ranges from excellent (3) to extremely bad (-3), the second from very much better (-3) to worse (1) without hypothyroidism. The 18-item main questionnaire asks for impact of hypothyroidism on various domains of life such as work life or sex life (see [25] for a complete list of domains). Nine of the domains have a “not applicable” option to guarantee individual relevance of the items. For each domain, if applicable, respondents rate whether life in this domain would be very much better (-3) to worse (1) without hypothyroidism. In addition, respondents rate the importance of the respective domain from very important (3) to not at all important (0). A weighted domain impact score is calculated by multiplying both ratings for each domain resulting in scores ranging from –9 (maximal negative impact of hypothyroidism on quality of life) to 3 (maximal positive impact of hypothyroidism). These scores can be summed into an Average Weighted Impact Score (AWI–18, ranging from –9 to 3) by summing all domain weighted impact scores and dividing the result by the number of applicable and completed domains if at least half of the items are applicable and completed. In addition, the AWI–14 can be calculated by excluding four items which overlap with the ThySRQ. Finally, a free comments section at the end allows respondents to indicate further domains not covered in the questionnaire.

The 15-item ThySRQ requires a yes/no response on whether each of the given hypothyroidism-related symptoms such as feeling cold or weight gain has been experienced in recent weeks (see [25] for a complete list of symptoms). If experienced, the amount of bother from the symptom is rated from not at all bothered (0) to very much bothered (3). If a symptom is not experienced bother rating for this item is zero.

The 7-item ThyTSQ covers several aspects of current treatment satisfaction, e.g. general satisfaction with or convenience of treatment (see [24] for a complete list of items). Items range from very satisfied (6) to very dissatisfied (0) or equivalent. Range of the summed score is 0–42. The ThyTSQ also offers a free comment section at the end.

### Patients and procedures

For the validation study 230 envelopes with questionnaires were given out by local endocrinologists and the outpatient department of the Clinic for Endocrinology at University Hospital Leipzig. The questionnaires were accompanied by a motivating letter and a questionnaire on basic sociodemographic and disease details as shown in Table 1.

**Table 1:** Characteristics of the sample for psychometric validation

**Sample characteristics**	**Mean**	**SD**	**Range**	**N**
Age (years)	43.5	16.3	19-75	101
Sex (female/male)	93/8			101
Marital Status	Single/Married/Divorced/Widowed			101
	37/48/12/4			
TSH (mU/l)	2.20	3.95	0.02-35.0	90
Treatment duration (years)	7.48	6.94	0.2 - 39.0	96
Current dose LT4* (μg/day)	97.9	42.4	0-250	99
Cause of hypothyroidism	Autoimmune thyroiditis			75
	Thyroidectomy for cold nodules or nodular goiter			13
	Ablative treatment for hyperthyroidism			7
	Lithium treatment			1
	Thyroidectomy – cause not specified			2
	Missing			3

*two participants additionally received 7.5/10 μg T3 respectively **comorbid conditions with potential influence occurring in more than one participant: depression (4), migraine (4), diabetes (4), asthma (3), heart disease (2), polycystic ovary syndrome (2) and pregnancy (2); several patients had multiple comorbid conditions. None of the non-reported rare comorbidities was a psychiatric illness. Abbreviaton: SD – Standard Deviation.

In the clinical study 25 patients with treated hypothyroidism due to autoimmune thyroiditis independent of the validation sample as well as 27 healthy control subjects were enrolled after written informed consent. Patients were recruited via internet advertisement, local endocrinologists and the MPI’s volunteer database. Healthy control subjects were recruited via the database alone. The database consists of volunteers recruited via the MPI’s website or via advertisement for former non-clinical studies. All participants were reimbursed for their time. The research protocol of both studies was approved by the ethics committee of the University of Leipzig and was in accordance with the latest version of the Declaration of Helsinki.

Patients in the clinical study filled in the three newly translated questionnaires ThySRQ, ThyDQoL and ThyTSQ alongside well-known and validated generic instruments of mood and well-being as part of a larger study. Healthy control subjects completed the same questionnaires except for the ThyTSQ and most parts of the ThyDQoL because they are not meaningful to healthy subjects. The ThyTSQ explicitly asks questions about the satisfaction with the treatment that healthy subjects do not receive and the ThyDQoL asks for the quality of life in several domains in the style “If I did not have underactive thyroid, my working life would be…”. However, the first question of the ThyDQoL asks for general quality of life and is thus meaningfully answerable for healthy control subjects. The ThySRQ asks for symptoms independent of the disease, e.g. “Have you felt tired in recent weeks?” which is also meaningful to healthy control subjects. Please note however that the use of any of the Thy questionnaires in a healthy population has not been validated yet and our study can thus only provide preliminary results. General perceived physical and mental health status was measured by the Short Form-36 (SF-36, [30]). Higher values in the range of 0–100 in the two subscales physical and mental health stand for better perceived health. General mental strain was assessed by the Symptom Check List (SCL-90-R, [31]), higher values in the range of 0–4 meaning higher strain. General well-being was measured with the 12-item Well-Being Questionnaire (WBQ-12, [32]), higher values in the range of 0–36 indicate better well-being. Generic instruments were given first to prevent their interpretation being influenced by the content of the disease-specific instruments. Depression was assessed by a questionnaire, the Beck Depression Inventory (BDI, [33]) and a structured interview, the Hamilton Depression Scale (HDS, [34]), in both instruments higher values mean greater amounts of depression. Cut-off scores for clinical depression are eleven points for the BDI and seven points for the HDS.

Basic sociodemographic data were asked for in written form. In addition, fasting blood samples were taken in the morning to assess TSH, fT3, fT4, TPOAb and TgAb. Blood samples were analysed at the Institute for Laboratory Medicine of the University Hospital Leipzig by the fully automated Roche cobas system (Roche, Basel, Switzerland).

### Statistical analysis

All questionnaire items entering psychometric evaluation were checked for normal distribution by investigating histograms and skewness scores. Normality as checked by histograms and Kolmogorov-Smirnov tests for normal distribution was not given for most variables. The skewness threshold of ±2.58 [26] was slightly exceeded by two items (ThyDQoL item getting out -3.2, ThySRQ item appetite 3.9). Reflect and log transformation did not significantly reduce skewness in these variables (getting out 2.7, appetite 3.7), so that statistics were calculated on the original data, but nonparametric tests were chosen for statistical analyses. Spearman’s rho and Mann–Whitney U-tests (MW-U-tests) were chosen for correlations and group comparisons respectively and parametric tests were employed for exploratory analyses only when no comparable nonparametric test was available, such as partial correlation or analysis of covariance (ANCOVA). Chi-square tests were used for comparison of nominal data. Statistical significance was accepted if *p* < 0.05 for all analyses and Bonferroni correction for multiple testing was applied where necessary.

## Results

### Sample evaluation study

102 questionnaires were returned anonymously in stamped addressed envelopes to the first author between September 2011 and November 2012; the response rate was 44%. Due to the anonymous design of the study no data were available on the non-responders. One participant reported not being treated yet and was thus excluded from any analysis. Among those reporting TSH values, 68 were within the normal range of 0.4-4.0 mU/l ([35], see Table 1). Seventy-one participants reported no or only symptom-free comorbid conditions such as hypertension. Thirty reported comorbidities likely to influence results (see Table 1) and subgroup comparisons were controlled for these influencing comorbidities.

### Psychometric evaluation of the newly translated instruments

As numbers for not applicable options were high in the ThyDQoL (0% for family to 45% for depression) they were coded as zero for factor and reliability analyses but as not applicable for calculation of the AWI-18 and AWI-14. One participant did not fill in the ThyDQoL, for all others the AWI-18 and AWI-14 could be calculated. Completion rate in the remaining sample was between 95 and 100% per item. Genuinely missing items were excluded for factor and reliability analysis, leaving 79 complete datasets for analysis. Unforced factor analysis on the 18 weighted-impact scores produced 4 factors, but the Varimax rotated factor loadings did not allow a meaningful interpretation. The screeplot suggested a single most important factor explaining 45% of the variance. A forced one-factor solution revealed that all variables loaded saliently (above 0.30) on the single factor, ranging from 0.36 (weight) to 0.82 (friendship and physical ability) and all but the weight item even above 0.40, implying robust findings [25]. Cronbach's alpha was 0.92, and all variables had an acceptable corrected item-total correlation above 0.20, ranging from 0.35 (weight) to 0.78 (physical ability). Thirty respondents used the free comments section. Eleven mentioned comorbidities not included in the present questionnaire. Six of forty-nine, or 12% of women of childbearing age (18–40 years) mentioned involuntary infertility. Fertility may thus be a potentially relevant domain for inclusion in future versions of the ThyDQoL at least when targeting younger women.

98 complete datasets were available for factor and reliability analysis of the ThySRQ. Two participants missed one item each and one participant missed the final page (four items). Unforced factor analysis on the symptom bother ratings revealed five factors. However, the factors could not be interpreted in a meaningful way and the screeplot pointed towards a single factor solution. The forced 1-factor solution explained 28% of the variance and variables loaded between 0.20 (constipation) and 0.74 (concentration). Only constipation loaded below 0.30 and nails (0.36) below 0.40 on the single factor. Cronbach's alpha was 0.81. All variables except for constipation (0.13) had a corrected item-total correlation above 0.20 ranging from 0.28 (nails) to 0.63 (concentration).

99 complete datasets were available for factor and reliability analysis of the ThyTSQ. One participant missed a single item and one the whole questionnaire. Unforced factor analysis produced a single factor as also suggested by the screeplot. This factor explained 57% of the variance. All variables loaded robustly (above 0.40) on this factor, ranging from 0.48 (convenience) to 0.89 (how well working). Cronbach's alpha was 0.86. All variables had a corrected item-total correlation above 0.20, ranging from 0.37 (convenience) to 0.80 (how well working). Twenty-one participants used the free comments section, but did so only to stress points already covered by the ThyTSQ.

### Descriptive results and comparison to the English originals

The current sample (n = 101) was significantly younger than the original sample (mean = 44 vs. 55 years, one-sample *t*-test *p* < 0.001; all comparison data from [25]) but comparable in the distribution of comorbidities. The present sample mean of the AWI-18 (n = 100) was -1.50 (SD = 1.4, range -5.7 to 0), indicating negative impact of hypothyroidism on quality of life. People reported significantly less impact on quality of life than in the original sample (mean = -3.11; *p* < 0.001). General quality of life was rated as “good” (n = 100, mean = 0.91, SD = 0.95, range -2 to +3) not significantly different from the original sample mean = 0.89 (*p* > 0.1) and general impact of hypothyroidism on quality of life was rated as “a little better without hypothyroidism” (n = 98, mean = -0.88, SD = 0.76, range -3 to 0 vs. mean = -1.25; *p* < 0.001), less negative than in the original sample. Mean number of reported symptoms was 5.6 (n = 98, SD = 3.3, range 0–13), which is significantly less than in the original sample (mean = 7.4; *p* < 0.001). Sample mean of the TSQ sum score was 31.6 (n = 99, SD = 7.6, range 10–42), comparable to the reference finding [25] (mean = 32.5; *p* > 0.1).

### Intercorrelations

Correlations were performed between the three new questionnaires to investigate construct validity. For better comparability between correlations, data were excluded listwise, resulting in n = 91 complete datasets entered into all analyses reported in Table 2.

**Table 2:** Spearman correlations between the ThyDQoL, ThyTSQ, and ThySRQ items

**ThyDQoL indices**	**ThyDQoL AWI-18**	**ThyDQoL AWI-14**	**ThyDQoL Present QoL**	**ThyDQoL Impact on QoL**	**ThyTSQ sumscore**
AWI-18	-----	0.97*	0.51*	0.75*	0.57*
AWI-14	-----	-----	0.52*	0.72*	0.56*
Present QoL	-----	-----	-----	0.30*	0.48*
Impact on QoL	-----	-----	-----	-----	0.55*
ThySRQ symptom bother ratings					
Tiredness	-0.39**	-0.39**	-0.28**	-0.34**	-0.37**
Weight gain	-0.38**	-0.33**	-0.14 n.s.	-0.34**	-0.27*
Cold	-0.23*	-0.20 n.s.	-0.27*	-0.14 n.s.	-0.07 n.s.
Constipation	-0.13 n.s.	-0.09 n.s.	-0.08 n.s.	-0.18 n.s.	-0.16 n.s.
Hair	-0.29*	-0.32**	-0.27*	-0.24*	-0.26*
Skin	-0.35**	-0.35**	-0.26*	-0.31**	-0.26*
Nails	-0.31**	-0.31**	-0.23*	-0.34**	-0.21 n.s.
Appetite	-0.25*	-0.23*	-0.19 n.s.	-0.11 n.s.	-0.02 n.s.
Hearing	-0.14 n.s.	-0.12 n.s.	-0.08 n.s.	-0.13 n.s.	0.03 n.s.
Voice	-0.32**	-0.30*	-0.30**	-0.05 n.s.	-0.21*
Speech	-0.37**	-0.35**	-0.22*	-0.25*	-0.13 n.s.
Memory	-0.22*	-0.20 n.s.	-0.22*	-0.20 n.s.	-0.06 n.s.
Concentration	-0.42**	-0.43**	-0.47**	-0.26*	-0.43**
Giddy	-0.33**	-0.32**	-0.23*	-0.31**	-0.22*
Depressed	-0.48**	-0.46**	-0.56**	-0.27*	-0.45**

*p < 0.05, **p < 0.0033 corrected for multiple testing within ThySRQ symptom bother ratings. Abbreviations: ThyDQoL – Thyroid-Dependent Quality of Life Questionnaire, ThySRQ – Underactive Thyroid Symptom Rating Questionnaire, ThyTSQ – Thyroid Treatment Satisfaction Questionnaire, AWI – Average Weighted Impact Score, QoL – Quality of Life.

AWI-18 and AWI-14 showed a similar correlation pattern to the ThySRQ items, so that spurious intercorrelations by overlapping items between AWI-18 and ThySRQ can be ruled out and the original AWI-18 will be used for all further analyses. The single-item impact on QoL is highly correlated with the multiple-item AWI-18, so that we will use only the AWI-18 for further analyses. Correlations between both AWI scores and ThySRQ symptom bother ratings ranged between -0.09 and -0.48 all in the expected direction but of small to moderate size, implying that symptom bother and impact on quality of life are related but distinct constructs. Treatment satisfaction as measured by the ThyTSQ showed low to moderate mainly negative correlations with the ThySRQ symptom bother ratings (range 0.03 to -0.45) and moderate positive correlations (range 0.48 to 0.57) with the different ThyDQoL indices. Treatment satisfaction is thus meaningfully related to but separable from the other two disease-specific PROMs.

Forward models of multiple regression analysis revealed that among those ThySRQ items correlating significantly with present QoL, depression (F(1,97) = 42.5, *p* < 0.001) and feeling cold (F(2,96) = 25.1, *p* < 0.001) significantly predicted present QoL, explaining 34% of the variance. The impact of hypothyroidism on quality of life (AWI-18) was significantly predicted by depression (F(1,97) = 33.9), speech problems (F(2,96) = 33.4), nail problems (F(3,95) = 27.0) and tiredness (F(4,94) = 22.3), explaining 49% of the variance, all *p* < 0.001. Analogously, depression (F(1,96) = 24.2) and tiredness (F(2,95) = 15.7) significantly predicted treatment satisfaction, explaining 25% of the variance, all *p* < 0.001. The ThySRQ depression item alone explained 31% of the variance of present QoL, 26% of variance of the impact of hypothyroidism on QoL and 20% of the variance of treatment satisfaction.

### Subgroup analyses

Subgroup analyses were performed with pairwise deletion of missing items in order to reduce information loss for the smaller sample sizes considered here. Within the group of adequately treated patients (n = 68) symptom bother ratings of depression (*r* = 0.47, *p* < 0.001) and hair (*r* = 0.37, *p* < 0.002) correlated significantly with TSH, depression even after Bonferroni correction (*p* < .0033). Additionally, age correlated negatively with TSH (*r* = -0.39, *p* < .002). Therefore, an exploratory partial correlation controlling for age was performed yielding the exact same pattern as above, depression (*r* = 0.44, *p* < 0.001) and hair (*r* = 0.42, *p* < 0.001) bother ratings correlating significantly even after Bonferroni correction. Similar results were obtained when considering either the full sample with available TSH values (n = 91) or only adequately treated patients with autoimmune thyroiditis (n = 51). Again depression was the only correlation surviving Bonferroni correction (*r* = 0.32, *p* < 0.002) in the full sample or just missing Bonferroni correction in the group with autoimmune thyroiditis (*r* = 0.37, *p* < 0.009).

Comparing adequately treated patients split by median TSH (n = 34 in both groups, TSH median = 1.44 mU/l) revealed that patients in the lower range were older (49 vs. 40 years; *p* < 0.05) but groups did not differ in the amount of comorbidities (*p* > 0.1), explicitly depression (n = 2 in each group) nor gender (p > 0.1) or type of diagnosis (autoimmune vs. other *p* = 0.1). Those in the upper TSH range reported significantly more bother by hair (1.3 vs. 0.5; *p* < 0.006), depression (1.4 vs. 0.6; *p* < 0.003) and concentration (1.2 vs. 0.7; *p* < 0.05) symptoms and overall more symptoms in the ThySRQ (6.4 vs. 4.8; *p* < 0.05). Exploratory ANCOVAs controlling for age revealed that only hair (*p* < 0.007) and depression symptom bother (*p* < 0.005) differed significantly between TSH groups independently of age. After Bonferroni correction, only depression symptom bother was significant before controlling for age.

Among the adequately treated participants with known cause of hypothyroidism those with autoimmune thyroiditis (n = 51) were compared to those reporting a different diagnosis (n = 16). Groups did not differ in gender and comorbidities, explicitly not in depression (n = 2 in each group), but in TSH (autoimmune 1.63 mU/l vs. other 1.05 mU/l; *p* < 0.01) and age (40.9 vs. 57.3 years; *p* < 0.001). The group with autoimmune thyroiditis reported significantly higher bother ratings for depression (1.22 vs. 0.33; *p* < 0.009) as well as reduced treatment satisfaction (30.7 vs. 35.4; *p* < 0.05) before Bonferroni correction (corrected *p* < .0033). In an exploratory ANCOVA with age and TSH as covariates the group effect on depression remained significant on an uncorrected level.

### Clinical sample

Two of the original 25 treated patients did not complete the study for reasons unrelated to disease status. Five of the remaining 23 treated patients had to be discarded because of unstable doses or persistently heightened TSH levels. Autoimmunity was either proven by positive TPOAb and/or TgAb or hypoechogenicity at thyroid ultrasound from clinical records. Nine of the originally 27 healthy control subjects had to be discarded for either positive TPOAb, TgAb or heightened TSH levels in two consecutive blood samples taken about 3 months apart, leaving 18 participants in both groups, see Table 3.

**Table 3:** Characteristics of the clinical sample with treated hypothyroidism in proven autoimmune thyroid disease and the healthy control group

**Sample characteristics**	**Treated patient group n = 18**			**Healthy control group n = 18**			***p*-value**
	**Mean**	**SD**	**Range**	**Mean**	**SD**	**Range**	
Age (years)	32.2	9.60	[18–54]	32.1	8.90	[18–53]	>0.1
Sex (female/male)	16/2			16/2			>0.1
TSH (mU/l)*	1.96	1.42	[0.2 - 5.3]	2.63	0.95	[0.9 - 5.0]	<0.05
fT3 (pmol/l)*	4.28	0.62	[3.2 - 5.6]	4.72	0.83	[3.2 - 6.3]	=0.07
fT4 (pmol/l)*	18.2	1.61	[14–21]	15.6	2.24	[14–23]	<0.001
TPOAb (U/ml)*	140	132	[9–457]	10.2	2.14	[7–14]	<0.001
TgAb (U/ml)*	313	604	[5–2424]	17.3	7.47	[5–36]	<0.001
Treatment duration (years)	4.41	4.21	[1–15]	not applicable			
Current dose (μg/day)	96.5	29.6	[50–150]				

*laboratory values of initial blood sample, reference range was 0.4-4.0 for Thyroid Stimulating Hormone (TSH), 3.1-6.79 for free Triiodothyronine (fT3), 12.8-20.4 for free Thyroxine (fT4), < 34 for Thyroid Peroxidase Antibodies (TPOAb) and < 33 for Thyroglobulin Antibodies (TgAb) [4,35]. Patients with slightly altered laboratory values that spontaneously recovered to the normal range in a second blood sample taken 3–6 months later were considered as thyroid healthy or adequately treated and included into the analysis. Abbreviaton: SD – Standard Deviation.

Correlations between the newly translated hypothyroidism-specific questionnaires and previously validated and published questionnaires on mood and well-being revealed that all correlated in the expected direction although only a subset reached significance, see Table 4. Correlations are between small and moderate.

**Table 4:** Spearman correlations between hypothyroidism-specific and generic patient-reported outcome measures in treated patients

**Specific vs. generic instruments**	**ThyDQoL present QoL**	**ThyDQoL AWI-18**	**ThySRQ symptom number**	**ThyTSQ sumscore**
Short Form-36 physical	0.40	0.33	-0.15	0.33
Short Form-36 mental	0.29	0.30	-0.39	0.31
WBQ-12	0.53*	0.64*	-0.61*	0.47
SCL-90-R	-0.48*	-0.53*	0.58*	-0.51*
Hamilton Depression Scale	-0.53*	-0.49*	0.45*	-0.74**
Beck Depression Inventory	-0.56*	-0.62*	0.29	-0.61*

*p < 0.05, **p < 0.002 Bonferroni corrected. Abbreviations: WBQ – Well-Being Questionnaire, SCL – Symptom Check List, ThyDQoL – Thyroid-Dependent Quality of Life Questionnaire, ThySRQ – Underactive Thyroid Symptom Rating Questionnaire, ThyTSQ – Thyroid Treatment Satisfaction Questionnaire, AWI – Average Weighted Impact Score, QoL – Quality of Life.

### Comparison of clinical groups

ThyDQoL and ThyTSQ are only meaningful to treated patients as explained above. In a clinical study including a control group only the ThySRQ and the ThyDQoL present QoL item are thus meaningful for group comparisons, although their use in a healthy sample still needs independent evaluation and reported results here are of preliminary nature. Comparison of the two groups in the clinical study revealed several significant results or statistical trends, see Table 5. Among the ThySRQ symptom bother ratings only tiredness (*p* < 0.003) reached significance after Bonferroni correction. Results for the other ThySRQ symptoms are not shown.

**Table 5:** Patient-reported outcome measures in patients with autoimmune thyroid disease and matched healthy control subjects

**Patient-reported outcomes**	**Treated patient group n = 18**		**Healthy control group n = 18**		***p-*** **value**
	**Mean**	**SD**	**Mean**	**SD**	
ThySRQ symptom number*	5.44	2.83	2.93	2.63	<0.05
ThyDQoL Present QoL	1.17	0.92	1.61	0.78	n.s.
Hamilton Depression Scale	6.00	3.76	3.72	2.35	=0.06
Beck Depression Inventory*	5.67	4.65	3.00	3.55	<0.05
Short Form-36 physical	54.80	8.50	56.07	5.07	n.s.
Short Form-36 mental	42.93	11.14	49.28	6.81	=0.09
WBQ-12	23.72	5.36	27.78	4.44	<0.05
SCL-90-R	0.39	0.28	0.35	0.33	n.s.
ThySRQ tiredness*	1.67	0.91	0.60	0.63	<0.0033

*Only available in n = 15 healthy control subjects, excluding the same subjects for all analyses does not change any of the results. Abbreviations: SD – Standard Deviation, WBQ – Well-Being Questionnaire, SCL – Symptom Check List, ThyDQoL – Thyroid-Dependent Quality of Life Questionnaire, ThySRQ – Underactive Thyroid Symptom Rating Questionnaire, QoL – Quality of Life.

### Correlation with thyroid parameters

Higher fT4 in the group of treated patients was correlated significantly with lower impact of hypothyroidism on quality of life as measured by AWI-18 (*r* = 0.51) before Bonferroni correction. None of the other thyroid parameters correlated significantly with the questionnaire data. However, in an exploratory inspection of the full correlation table, the majority of correlations were in the expected direction of more negative reports with worse thyroid status for TSH, fT4 and TPOAb and between the generic instruments and TgAb, see Table 6. Interestingly, for the disease-specific questionnaires, this pattern was completely reversed for fT3 and TgAb.

**Table 6:** Spearman correlations between laboratory values and patient-reported outcomes in the group of treated patients

**Laboratory results - patient-reported outcomes**	**TSH**	**fT3**	**fT4**	**TPOAb**	**TgAb**
ThyDQoL AWI-18	-0.23^1^	-0.04^2^	0.51^1**^	-0.19^1^	0.07^2^
ThyDQoL Present QoLresent QoL	-0.23^1^	-0.28^2^	0.30^1^	-0.01^1^	0.19^2^
ThySRQ number symptoms	0.26^1^	0.09^2^	-0.31^1^	0.15^1^	-0.21^2^
ThyTSQ	-0.17^1^	-0.39^2^	0.39^1^	-0.02^1^	0.05^2^
Short Form-36 physical	-0.37^1^	-0.07^2^	0.21^1^	-0.33^1^	-0.04^1^
Short Form-36 mental	0.16^2^	-0.21^2^	-0.08^2^	0.27^2^	0.20^2^
SCL-90-R	0.05^1^	0.02^2^	-0.23^1^	0.24^1^	0.24^1^
WBQ-12	-0.15^1^	0.05^1^	0.36^1^	-0.12^1^	-0.03^1^
Hamilton Depression Scale Scale	0.19^1^	0.10^2^	-0.40^1^	0.43^1^	0.27^1^
Beck Depression Inventory	-0.12^2^	-0.16^1^	-0.40^1^	0.06^1^	0.16^1^

*p < 0.05, ^1^in the expected direction, ^2^against the expected direction. Abbreviations: WBQ – Well-Being Questionnaire, SCL – Symptom Check List, ThyDQoL – Thyroid-Dependent Quality of Life Questionnaire, ThySRQ – Underactive Thyroid Symptom Rating Questionnaire, ThyTSQ – Thyroid Treatment Satisfaction Questionnaire, AWI – Average Weighted Impact Score, QoL – Quality of Life, TSH – Thyroid Stimulating Hormone, fT3 – free Triiodothyronine, fT4 – free Thyroxine, TPOAb – Thyroid Peroxidase Antibodies, TgAb – Thyroglobulin Antibodies.

## Discussion

### Validity of the newly translated questionnaires

All three questionnaires reached psychometric validity comparable to the English originals and most importantly, mostly reached the standards as laid out in [25] (factor loadings on single factor >0.4, Cronbach’s Alpha >0.8, Corrected item-total correlations >0.2). The ThySRQ only slightly missed the robustly salient factor loading for the items constipation and nail problems as similarly reported for the original ThySRQ [25] and the weight gain item from the ThyDQoL missed the robust 0.4 loading, but still loaded saliently. The current sample was on average 10 years younger than the original sample and reported slightly better status on ThyDQoL and ThySRQ, but similar treatment satisfaction in the ThyTSQ. However, the current sample still reported a negative impact of hypothyroidism and an average of six symptoms despite biochemically adequate treatment, which is in line with previous literature reporting residual complaints [6,7]. Treatment satisfaction was in the moderately positive range but with room for improvement.

Due to the anonymous design of the study, which allowed maximal data privacy and reduced bias due to social desirability effects, no data were available on the non-responders. However, our sample is comparable to similar questionnaire studies on PROMs in treated hypothyroidism, which include data on non-responders according to age, sex ratio, cause of hypothyroidism, percentage normal TSH levels, and disease duration. Moreover, comparison of responders to non-responders in these similar studies revealed only a slight bias with respect to age and sex ratio [6,25,26]. Furthermore, our sample showed a broad range and variance of disease parameters (see Table 1) and reported severity of impairment so that it is unlikely that the responders form a highly selective group within the population of treated patients with hypothyroidism.

Intercorrelation of ThySRQ items with ThyDQoL replicated previous findings [25] again showing the AWI-18 can be used together with the ThySRQ without causing spurious correlations by four shared items. We here additionally included the ThyTSQ and found comparable effect sizes of correlations to ThySRQ items and AWI-18 as already shown between the other two questionnaires, implying that the three questionnaires measure related but sufficiently separate constructs. Among the ThySRQ items, it was depression that explained most variance to predict present quality of life, impact of hypothyroidism on quality of life and treatment satisfaction, so depressive symptoms should be given special attention when evaluating patient reports.

Higher depression bother rating was robustly associated with higher TSH across adequately treated patients or the whole sample. Interestingly, although no further results reached significance, the large majority of correlations above 0.20 were in the direction of lower normal TSH values with better patient-reported outcomes. Our results are in line with the literature proposing to treat patients towards TSH levels in the lower normal range [5,28]. However, treatment outcomes other than patient reports have to be considered, such as effects on heart function or cognition [8,36], especially in patients beyond the age of 75 [37], in order to achieve optimal treatment for the individual patient. Patients reporting autoimmune thyroid disease as opposed to other causes for hypothyroidism reported significantly more bother by depressive symptoms, in line with claims of an independent role for autoimmunity in residual symptoms [9-11].

### Clinical study

Correlations between hypothyroidism-related questionnaires and generic instruments in the clinical sample were all in the expected direction. The effect size was small to moderate, meaning that the new disease-specific questionnaires do share common variance with the generic instruments but also hold distinct variance to the more generic instruments. They are thus valid additions for future studies. Those disease-specific questionnaires applicable also to healthy control subjects (ThySRQ and the present QoL item of ThyDQoL) proved to be as sensitive in detecting subtle differences in small clinical samples as the well-established generic instruments. It is remarkable that descriptively all results were in the expected direction of more negative patient reports in the treated patient group adding to the literature on residual symptoms despite biochemically adequate treatment [6,7].

Only a few correlations between hormone levels and patient-reported outcomes in treated patients reached significance, possibly due to the low power of the study. However, TSH, fT4 and TPOAb consistently correlated in the expected direction of more negative PROMs with worse biochemical status, and being most pronounced in the disease-specific questionnaires. This, though necessarily preliminary, favours a subtle relationship between certain biochemical thyroid hormone markers within the normal range and PROMs in treated patients. In contrast, fT3 and TgAb showed a reverse (fT3) or inconsistent (TgAb) relationship to PROMs, in line with clinical routine that does not consider them as valid markers of thyroid hormonal status. Interestingly, TPOAb were equally related to patient-reported outcomes as TSH and fT4. This is in line with literature considering autoimmunity as an independent factor for treatment outcome [10,27].

We acknowledge the small sample size of the clinical study. However, our sample size is comparable to other clinical studies [23] in the field and it is thus important to show that instruments are also sensitive enough to detect group differences in small samples as shown here. Among the Thy questionnaires, we found significant group differences for the number of reported symptoms in the ThySRQ and for the tiredness item, but not for the overall quality of life question in the ThyDQoL. These results should be interpreted with caution as the use of the questionnaires in healthy control subjects has not been validated so far, but are still of preliminary interest to encourage further validation and use of the questionnaires in cross-sectional studies including healthy control subjects. Our clinical sample was of a young age range in order to exclude effects of aging and comorbidities on the data, and thus does not contribute to research on possible specificities of hypothyroidism in the elderly such as beneficial effects on the coronary system in patients over 85 years, which leads to reduced mortality [37]. Therefore, treatment targets a slightly higher TSH value in the very elderly. Studies on quality of life and perceived symptoms in this age group are not known to us and would be difficult to interpret, because symptoms of hypothyroidism greatly resemble those of general aging and therefore are prone to confusion in elderly subjects [37]. Our findings therefore cannot be extended to the very elderly. Most importantly however, the 10-year age difference and stronger severity of reported symptoms between our evaluation sample and the original one [25] did not influence the findings on the psychometric properties of the questionnaires, so that our main finding, the sufficient psychometric quality of the questionnaires can be generalised to the use in young and middle aged cohorts.

Finally, the majority of participants were female, owing to the gender bias in the prevalence of the disease, as a consequence of which our results can only be generalised reliably to the female population and the study included only patients already on treatment for hypothyroidism, so that the interpretation is confined to the group of already treated patients.

## Conclusions

The three hypothyroidism-specific PROMs ThyDQoL, ThySRQ and ThyTSQ, introduced here as new German translations, show good psychometric properties and meaningful relations to TSH. In a small clinical sample we have found preliminary evidence for construct validity with well-established generic instruments and for a relationship to thyroid laboratory measures. We thus recommend them for use in clinical studies and encourage further linguistic validation into other languages to improve available research tools as a prerequisite for progress in clinical research on hypothyroidism.

## Abbreviations

ThyDQoL: Thyroid-Dependent Quality of Life Questionnaire; ThySRQ: Underactive Thyroid Symptom Rating Questionnaire; ThyTSQ: Thyroid Treatment Satisfaction Questionnaire; PROMs: Patient-reported outcome measures; TSH: Thyroid Stimulating Hormone; fT3: free Triiodothyronine; fT4: free Thyroxine; TPOAb: Thyroid Peroxidase Antibodies; TgAb: Thyroglobulin Antibodies.

## Competing interests

The authors declare that they have no competing interests.

## Access to questionnaires

For access to the questionnaires, visit http://www.healthpsychologyresearch.com.

## Authors’ contributions

EQ initiated and coordinated the linguistic validation, designed both studies, collected and analysed the data and wrote the manuscript. AV initiated the overall project. JK supervised laboratory analyses. SK recruited patients and supervised data analysis and drafting of the manuscript. All authors read and approved the final manuscript.
